# Resolutions of Respect on the Late Dr. William H. Atkinson, of New York

**Published:** 1891-09

**Authors:** 


### RESOLUTIONS OF RESPECT ON THE LATE DR. WILLIAM H. ATKINSON, OF NEW YORK.

AT a meeting of the Massachusetts Dental Society, the following resolutions were unanimously adopted:

WHEREAS, The dispensing hand of Divine Providence has removed from our midst a much-loved brother, Dr. William H. Atkinson, of New York, an honorary member of this Society, whose wisdom, learning, and skill called forth our highest appreciation. Therefore, be it

*Resolved*, That we tender to his afflicted relatives our sincere condolence and our earnest sympathy in their irreparable loss.

GEORGE F. EAMES,
R. R. ANDREWS,
STEPHEN G. STEVENS,
*Committee.*

